# Association Between Insulin-like Growth Factor-1 rs35767 Polymorphism and Type 2 Diabetes Mellitus Susceptibility: A Meta-Analysis

**DOI:** 10.3389/fgene.2021.774489

**Published:** 2021-11-22

**Authors:** Qiaoli Zeng, Dehua Zou, Qiaodi Zeng, Xiaoming Chen, Yue Wei, Runmin Guo

**Affiliations:** ^1^ Department of Internal Medicine, Shunde Women and Children’s Hospital (Maternity and Child Healthcare Hospital of Shunde Foshan), Guangdong Medical University, Foshan, China; ^2^ Key Laboratory of Research in Maternal and Child Medicine and Birth Defects, Guangdong Medical University, Foshan, China; ^3^ Shunde Women and Children’s Hospital (Maternity and Child Healthcare Hospital of Shunde Foshan), Matenal and Child Research Institute, Guangdong Medical University, Foshan, China; ^4^ State Key Laboratory for Quality Research of Chinese Medicines, Macau University of Science and Technology, Taipa, Macau (SAR) China; ^5^ Department of Clinical Laboratory, People’s Hospital of Haiyuan County, Zhongwei, China; ^6^ Department of Endocrinology, Affiliated Hospital of Guangdong Medical University, Zhanjiang, China; ^7^ Department of Ultrasound, Shunde Women and Children’s Hospital (Maternity and Child Healthcare Hospital of Shunde Foshan), Guangdong Medical University, Foshan, China

**Keywords:** type 2 diabete mellitus, insulin-like growth factor-1, rs35767, susceptibility, meta-analysis

## Abstract

**Background:** Insulin-like growth factor-1 (IGF-1) has been demonstrated to increase fatty acid *β* oxidation during fasting, and play an important role in regulating lipid metabolism and type 2 diabetes mellitus (T2DM). The rs35767 (T > C) polymorphism, a functional SNP was found in *IGF-1* promoter, which may directly affect *IGF-1* expression. However, the inconsistent findings showed on the *IGF-1* rs35767 polymorphism and T2DM risk.

**Methods:** We performed a comprehensive meta-analysis to estimate the association between the *IGF-1* rs35767 and T2DM risk among four genetic models (the allele, additive, recessive and dominant models).

**Results:** A total 49,587 T2DM cases and 97,906 NDM controls were included in the allele model, a total 2256 T2DM cases and 2228 NDM controls were included in the other three genetic models (the additive; recessive and dominant models). In overall analysis, the *IGF-1* rs35767 was shown to be significantly associated with increased T2DM risk for the allele model (T vs. C: OR = 1.251, 95% CI: 1.082–1.447, *p* = 0.002), additive model (homozygote comparisons: TT vs. CC: OR = 2.433, 95% CI: 1.095–5.405, *p* = 0.029; heterozygote comparisons: TC vs. CC: OR = 1.623, 95% CI: 1.055–2.495, *p* = 0.027) and dominant model (TT + CT vs. CC: OR = 1.934, 95% CI: 1.148–3.257, *p* = 0.013) with random effects model. After omitting Gouda’s study could reduce the heterogeneity, especially in the recessive model (TT vs. CC + CT: I^2^ = 38.7%, *p* = 0.163), the fixed effects model for recessive effect of the T allele (TT vs. CC + CT) produce results that were of borderline statistical significance (OR = 1.206, 95% CI: 1.004–1.448, *p* = 0.045). And increasing the risk of T2DM in Uyghur population of subgroup for the allele model.

**Conclusion:** The initial analyses that included all studies showed statistically significant associations between the rs35767 SNP and type 2 diabetes, but after removing the Gouda et al. study produced results that were mostly not statistically significant. Therefore, there is not enough evidence from the results of the meta-analysis to indicate that the rs35767 SNP has a statistically significant association with type 2 diabetes.

## 1 Introduction

Diabetes is one of the most common chronic metabolic disorder diseases in the worldwide, over 90% of the diabetes patients are type 2 diabetes mellitus (T2DM), which is characterized by insulin resistance in peripheral tissues and dysregulated insulin secretion by pancreatic beta (*β*) cells (Banerjee and Vats, 2014; Song et al., 2015). Substantial evidence suggests that insulin resistance, an inherited genetic defect, is the basis and major feature of T2DM (DeFronzo and Tripathy, 2009; Cai et al., 2019; Li et al., 2021). Insulin resistance is attributable to excess fatty acids and proinflammatory cytokines, which leads to impaired glucose transport and increases fat breakdown. Since there is an inadequate response or production of insulin, the body responds by inappropriately increasing glucagon, thus further contributing to hyperglycemia. Accumulated data have revealed that lipid abnormalities are associated with insulin resistance and contribute to T2DM (Johnson and Olefsky, 2013; Perry et al., 2014; Li et al., 2021). Studies have also revealed lipid metabolism-related genes and their single-nucleotide polymorphisms (SNPs) associated with insulin resistance and the development of T2DM (Ruchat et al., 2009; Dupuis et al., 2010; Chistiakov et al., 2012; Langberg et al., 2012; Mannino et al., 2013; Li et al., 2014; Thankamony et al., 2014; Yuan et al., 2015; Li et al., 2021).

*Insulin-like growth factor-1* (*IGF-1*) is a circulating growth factor which structure is highly homologous with pro-insulin. *IGF-1* is expresses in insulin-resistant tissue, it downregulates free fatty acid and increases fatty acid *β* oxidation during fasting (Thankamony et al., 2014; Li et al., 2021). It plays an important role in regulating lipid metabolism and insulin sensitivity (Seppä et al., 2015; Gouda et al., 2019), since it effects on glucose homeostasis and associated with insulin resistance (Li et al., 2011; Li et al., 2014; Dai et al., 2015; Ming et al., 2015; Yuan et al., 2015; Wei et al., 2018; Liao et al., 2019; Regué et al., 2019; Li et al., 2021). Previous studies have been reported that people with a low *IGF-1* level are prone to have diabetes mellitus (Chen et al., 2013; Colao et al., 2013; Shankar and Li, 2013). Polymorphisms in the *IGF-1* gene can directly affect *IGF-1* expression. The rs35767 (T > C) polymorphism, a functional SNP was found in *IGF-1* promoter, in which the promoter with C allele showed a higher transcriptional activity than promoter with T allele (Telgmann et al., 2009). Therefore, rs35767 may contribute to insulin resistance involving lipid metabolism in T2DM.

A significant association of *IGF-1* rs35767 with T2DM has been reported in several case-control studies (Gouda et al., 2019; Gulixiati·Maimaitituersun. 2020; Wang. 2019; Zhang et al., 2017; Song et al., 2015). However, seven studies failed to replicate the results (Dupuis et al., 2010; Hu et al., 2010; Fujita et al., 2012; Liu et al., 2012; Zhao et al., 2016; Li et al., 2019; Li et al., 2021). In veiw of the inconsistent results, whether *IGF-1* rs35767 is associated with T2DM remains to be determined. In this meta-analysis, we estimate the association of *IGF-1* rs35767 with T2DM among four different genetic models.

## 2 Materials and Methods

### 2.1 Literature Search

The Google Scholar, PubMed and Chinese National Knowledge Infrastructure were comprehensively searched for related studies published before July 31, 2021, using the key terms: “insulin-like growth factor 1 or *IGF-1* or *IGF1*,” “rs35767 or rs35767 (T > C) or rs35767 (A > G),” “polymorphism or SNP or mutation or variant” and “diabetes or type 2 diabetes or T2DM.” All searches had no language limitations. Eligible studies were estimated by reading full texts, and excluded substandard studies.

### 2.2 Inclusion and Exclusion Criteria

The following inclusion criteria: 1) case-control or cohort studies that relate to the *IGF-1* rs35767 and T2DM risk; 2) sufficient raw data or adequate data for assessing odds ratios (ORs) with corresponding 95% confidence intervals (CIs); and 3) The diagnostic standard of T2DM conformed to the World Health Organization.

The following exclusion criteria: 1) not a case-control study; 2) irrelevant to *IGF-1* rs35767 and T2DM risk; 3) lacking detailed data; and 4) control subjects is not in Hardy-Weinberg equilibrium (HWE).

### 2.3 Data Extraction

Data were independently extracted by two authors from the eligible studies and collected the following data: first author, year of publication, origin, the numbers of T2DM cases and NDM controls, gender and age, BMI (kg/m^2^), the distributions number of genotype and alleles, ORs with 95% CI, or ability to calculate the OR and 95% CI. *p*-value for the HWE of NDM controls.

### 2.4 Statistical Analysis

Statistical analyses using the STATA v.14.0 software (Stata Corporation, TX, United States). Four genetic models were evaluated in this meta-analysis: the allele model (T vs. C); the additive model (homozygote comparisons: TT vs. CC; heterozygote comparisons: TC vs. CC); the recessive model (TT vs. CC + CT) and the dominant model (TT + CT vs. CC). Using Q-test and I^2^ test to estimate the genetic heterogeneity. OR with corresponding 95% CIs were calculated by the random effectss model when *p* < 0.01 and I^2^ > 50% (He et al., 2015; Zhang et al., 2016). Otherwise, the fixed effectss model were used. Sensitivity analyses were implemented to evaluate the stability of the overall effect by excluding a study at a time. The Hardy-Weinberg equilibrium for the NDM controls was assessed using Pearson’s Chi-squared test. Using Bgger’s test to evaluate publication bias (Shen et al., 2015; Li et al., 2016; Liu et al., 2017; Han et al., 2019).

## 3 Results

### 3.1 Study Inclusion and Characteristics

**FIGURE 1:**
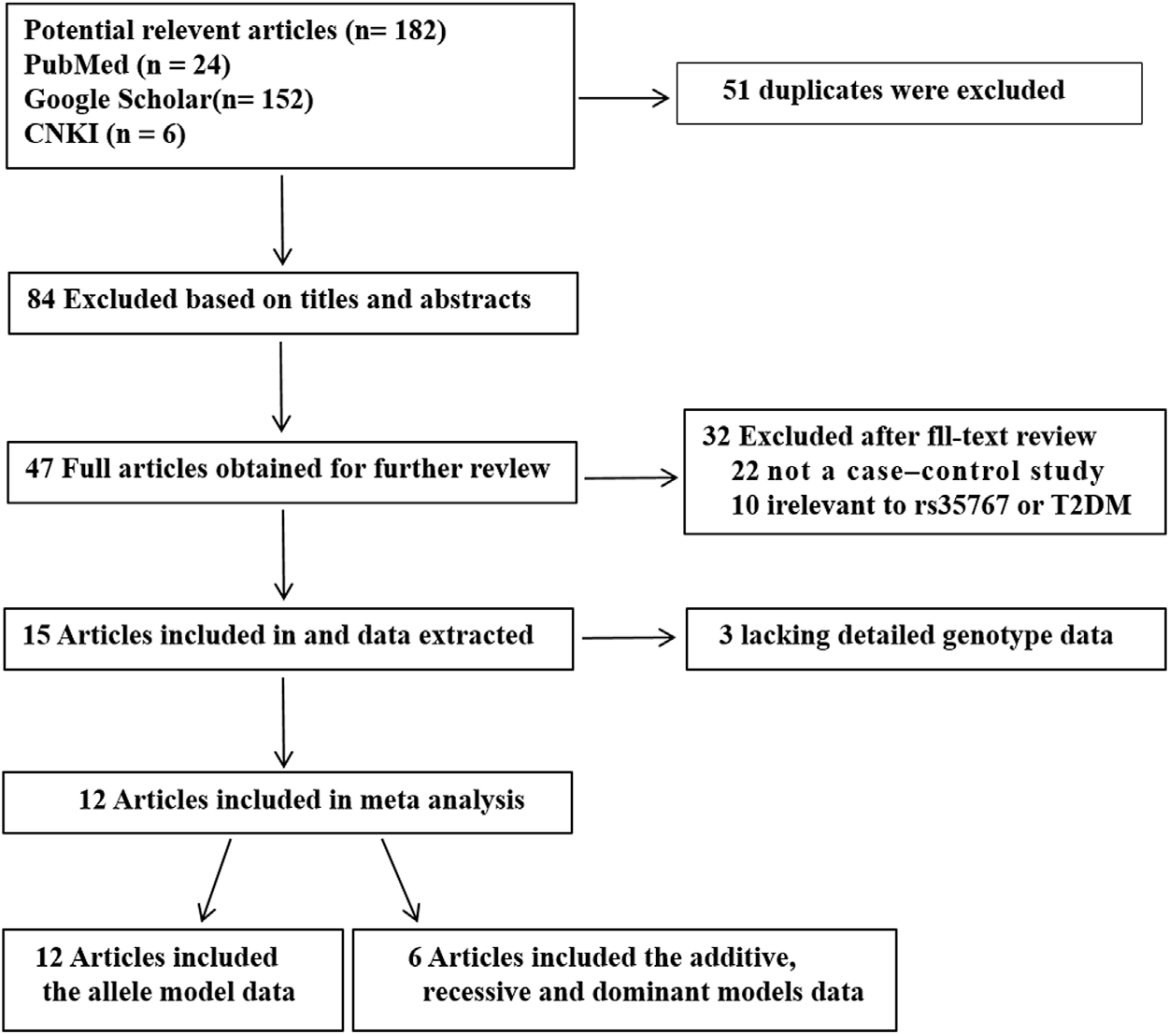
Flow chart of researches selection in the meta-analysis.

A total of 182 potential articles obtained through initial search. 51 duplicates were excluded. Then 131 studies were screened on title and abstract, 84 of them were excluded. The left 47 articles were evaluated by full-text reading, 35 of them were excluded cause that 22 were not case-control researchs, 10 were not related to rs35767 or T2DM, three did not provided sufficient data. 12 articles were included that there are six articles including five in English and one in Chinese just of the allele model data, and other six articles including four in English and two in Chinese of four genetic models data (the allele, additive, recessive and dominant models). Flow chart of researches selection in the meta-analysis was shown in Figure 1. A total 49,587 T2DM cases and 97,906 NDM controls were included in the allele model, a total 2256 T2DM cases and 2228 NDM controls were included in the other three genetic models (the additive; recessive and dominant models). The characteristics of each study are shown in Table 1 and Supplementary Table S1.

**TABLE 1:** Characteristics of each study included in this meta-analysis.

					Allele distribution				Genotype distribution					
					T2DM, n		NDM, n		T2DM, n			NDM, n		
Authors	Origin	Gender	T2DM/NDM, n	ORs with 95% CI (T vs. C)	C	T	C	T	CC	CT	TT	CC	CT	TT
Li et al. (2021)	Chinese (Yunnan)	M/F	1194/1274	0.928 (0.826−1.042)	1538	850	1597	951	513	512	169	500	597	177
Gulixiati et al. (2020)	Chinese (Xinjiang)	M/F	220/229	1.452 (1.092−1.931)	287	153	335	123	93	101	26	120	65	14
Gouda et al. (2019)	Egyptian	F	180/165	5.103 (3.641−7.153)	72	288	185	145	12	48	120	60	65	40
Wang et al. (2019)	Chinese (Tianjin)	M/F	367/367	1.322 (1.065−1.641)	460	274	506	228	146	168	53	176	154	37
Zhang et al. (2017)	Chinese (Hebei)	M/F	244/142	1.388 (1.025−1.879)	280	208	185	99	77	126	41	56	73	13
Song et al. (2015)	Chinese (Xinjiang)	M/F	51/51	1.900 (1.006−3.587)	67	33	81	21	21	25	4	34	13	4
Li et al. (2019)	Chinese (Tianjin)	F	80/1160	1.043 (0.727−1.494)	−	−	−	−	−	−	−	−	−	−
Zhao et al. (2016)	Chinese (Xinjiang)	M/F	130/135	1.480 (0.980-2.230)	−	−	−	−	−	−	−	−	−	−
Fujita et al. (2012)	Japanese	M/F	2632/2050	0.990 (0.912-1.075)	−	−	−	−	−	−	−	−	−	−
Liu et al. (2011)	Chinese (Beijing, Shanghai)	M/F	424/1899	0.935 (0.795-1.099)	−	−	−	−	−	−	−	−	−	−
Hu et al. (2010)	Chinese (Shanghai)	M/F	3410/3412	1.027 (0.956-1.103)	−	−	−	−	−	−	−	−	−	−
Dupuis et al. (2010)	European	M/F	40655/87022	0.962 (0.890-1.038)	−	−	−	−	−	−	−	−	−	−

n, Number; M, Male; F, Female; T2DM, type 2 diabetes mellitus; NDM, Non-diabetic subject;OR, odds ratio; CI, confidence interval; (-), not applicable.

### 3.2 Meta-Analysis

The association between the *IGF-1* rs35767 polymorphism and T2DM were evaluated using ORs and 95% CI in the allele model (12 studies, 49587 T2DM cases and 97906 NDM controls) and the additive; recessive and dominant models (6 studies, 2256 T2DM cases and 2228 NDM controls).

**FIGURE 2:**
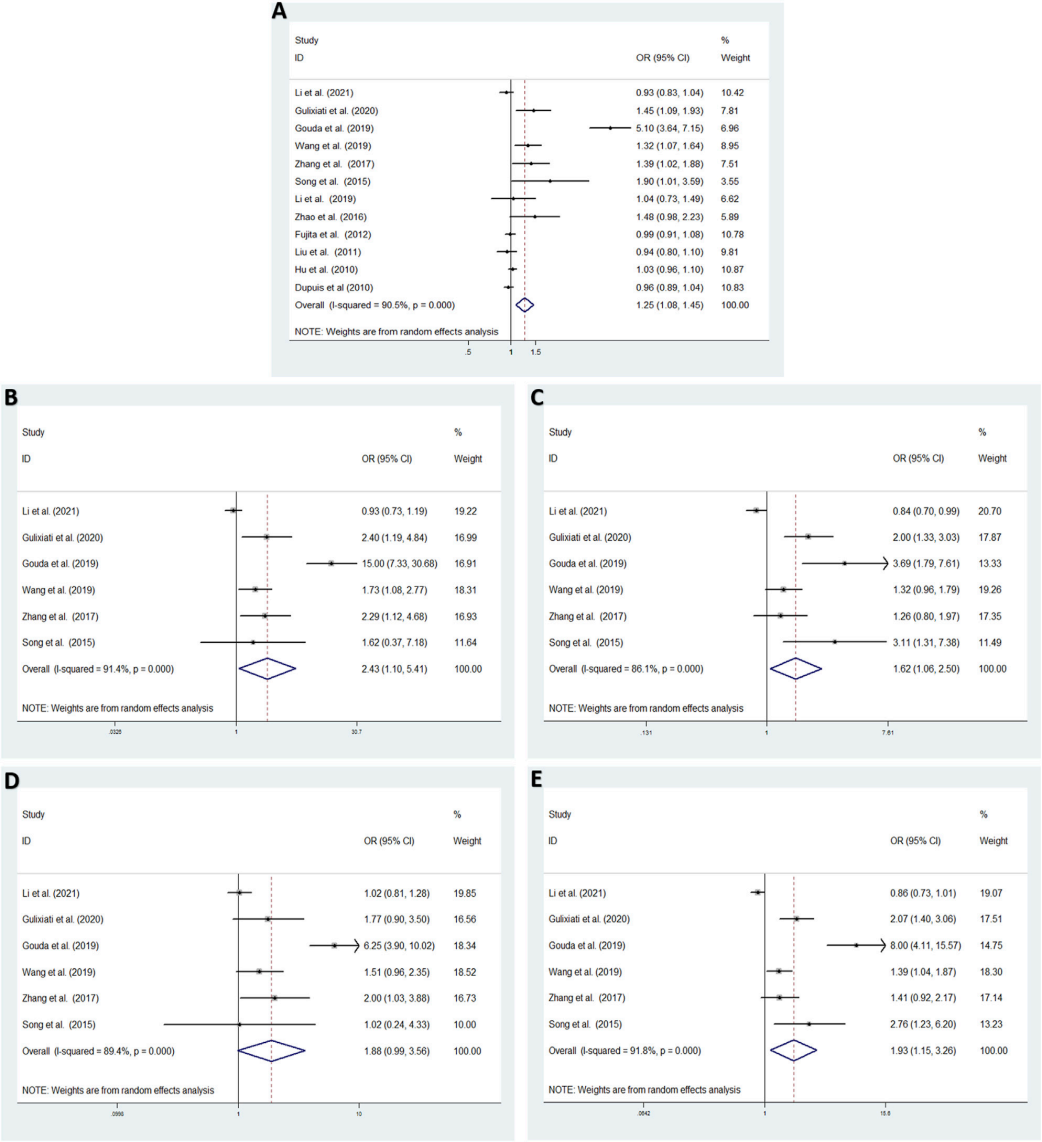
Meta-analysis with a random effects model for the association between the *IGF-1* rs35767 and T2DM susceptibility. **(A)** Allele model, T vs. C. **(B)** Additive model (homozygote comparisons): TT vs. CC.**(C)** Additive model (heterozygote comparisons): TC vs. CC. **(D)** Recessive model, TT vs. CC + CT.**(E)** Dominant model, TT + CT vs. CC. OR: odds ratio, CI: confidence interval, I-squared: measure to quantify the degree of heterogeneity in meta-analyses.

In overall analysis, A random effects model were used to analyze the allele, additive, recessive and dominant models. The *IGF-1* rs35767 was shown to be significantly associated with increased T2DM risk for the allele model (T vs. C: OR = 1.251, 95% CI: 1.082–1.447,*p* = 0.002), additive model (homozygote comparisons: TT vs. CC: OR = 2.433, 95% CI: 1.095–5.405, *p* = 0.029; heterozygote comparisons: TC vs. CC: OR = 1.623, 95% CI: 1.055–2.495, *p* = 0.027) and dominant model (TT + CT vs. CC: OR = 1.934, 95% CI: 1.148–3.257, *p* = 0.013). The results showed no significant difference for the recessive model (TT vs. CC + CT: OR = 1.876, 95% CI: 0.989–3.559, *p* = 0.054) (Figure 2).

### 3.3 Sensitivity Analysis

**FIGURE 3:**
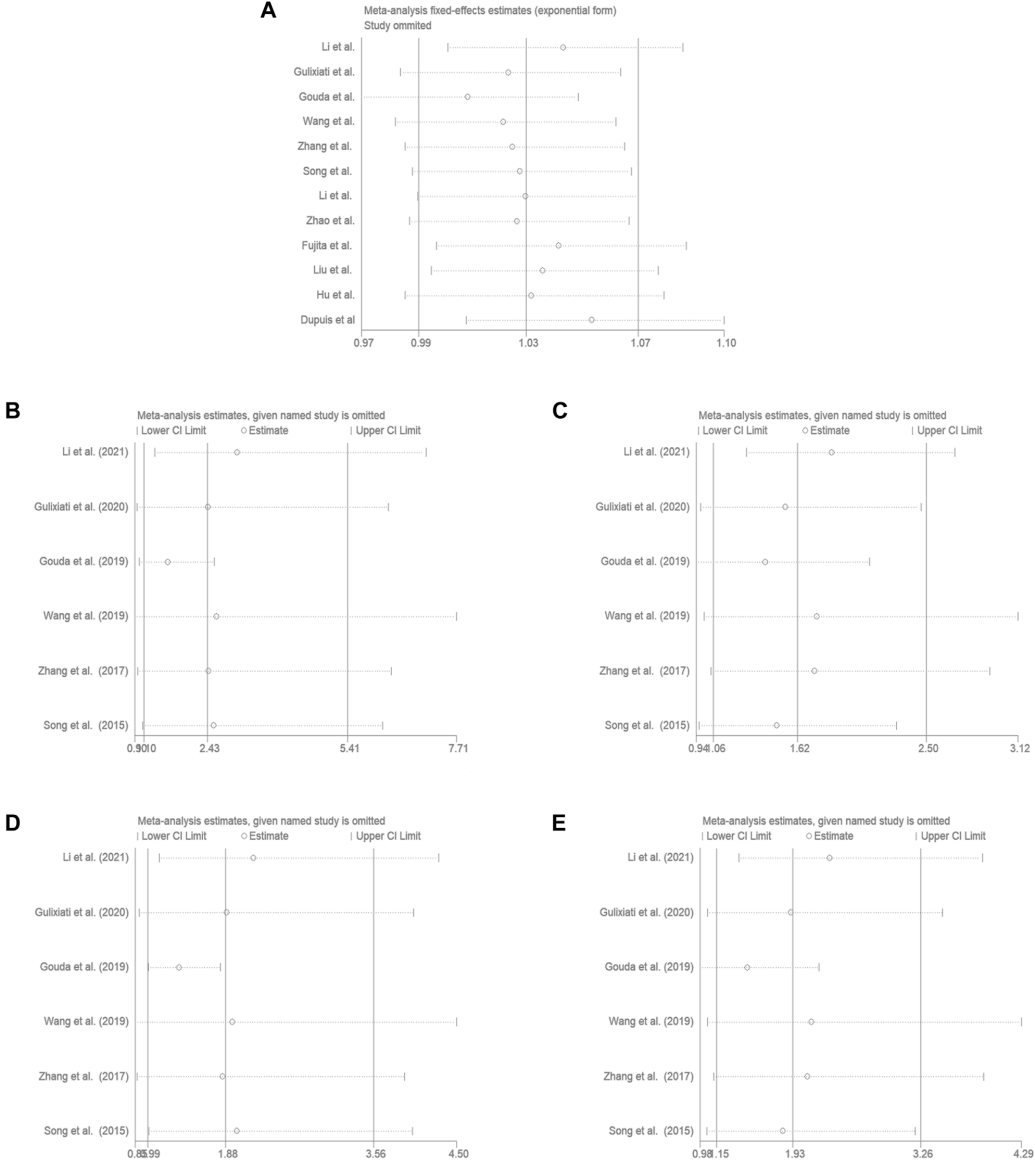
Sensitivity analysis by iteratively removing one study at a time. **(A)** Allele model, T vs. C. **(B)** Additive model (homozygote comparisons): TT vs. CC. **(C)** Additive model (heterozygote comparisons): TC vs. CC. **(D)** Recessive model, TT vs. CC + CT. **(E)** Dominant model, TT + CT vs. CC.

**FIGURE 4:**
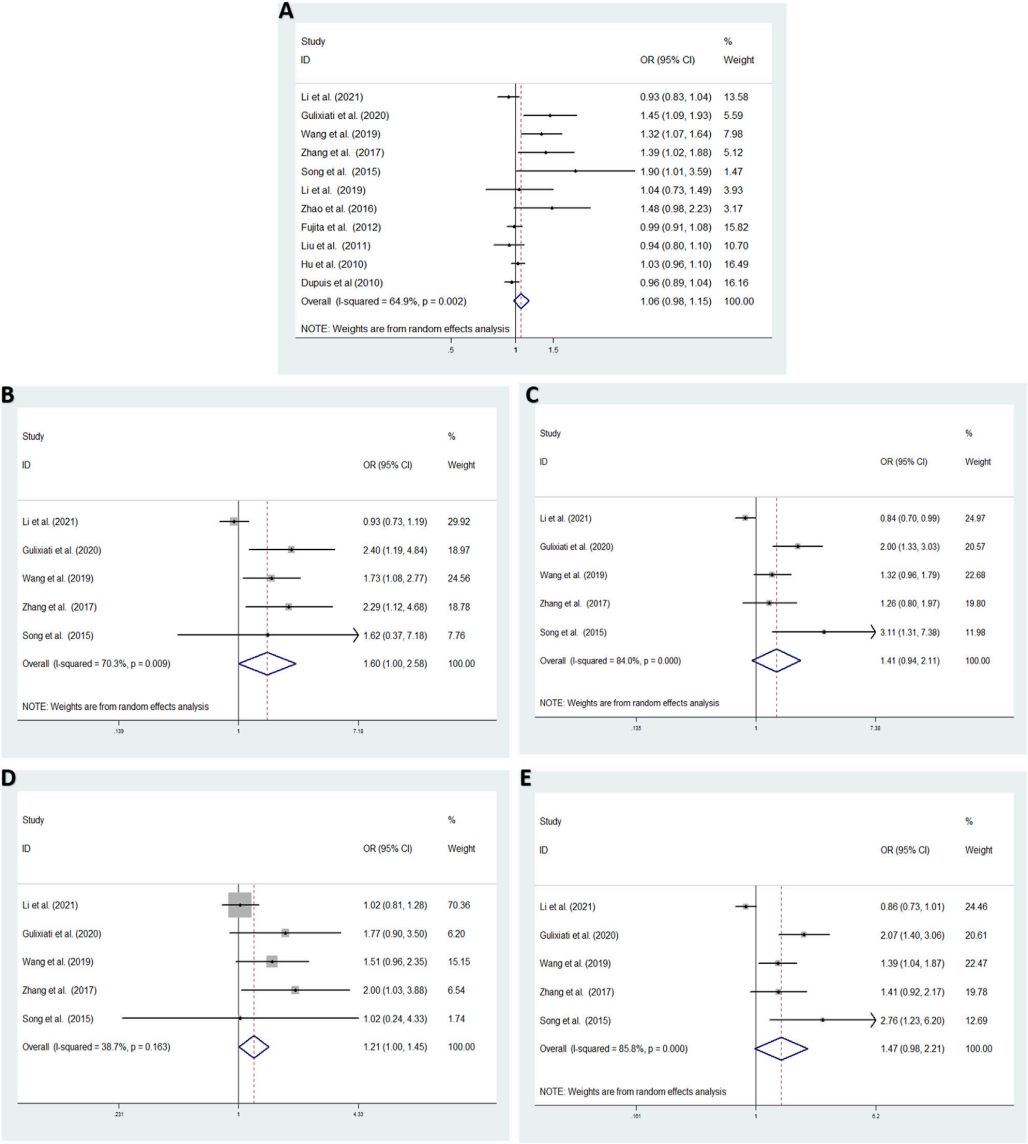
Meta-analysis for the association between the *IGF-1* rs35767 and T2DM susceptibility after omitting Gouda’s study. **(A)** Allele model, T vs. C (random effects model). **(B)** Additive model (homozygote comparisons): TT vs. CC. (random effects model). **(C)** Additive model (heterozygote comparisons): TC vs. CC (random effects model) **(D)** Recessive model, TT vs. CC + CT (fixed effects model) **(E)** Dominant model, TT + CT vs. CC (random effects model). OR: odds ratio, CI: confidence interval, I-squared: measure to quantify the degree of heterogeneity in meta-analyses.

Aiming to estimate the influence of each study on the overall OR below four genetic models and analysis the sources of high heterogeneity, sensitivity meta-analysis was performed with random effects model. The results were showed in Figure3, omitting Gouda’s study could reduce the heterogeneity, especially in the recessive model (TT vs. CC + CT: I^2^ = 38.7%, *p* = 0.163), the fixed effects model for recessive effect of the T allele (TT vs. CC + CT) produce results that were of borderline statistical significance (OR = 1.206, 95% CI: 1.004–1.448, *p* = 0.045); In addtion, other three modal show moderate degree of heterogeneity (T vs. C: I^2^ = 64.9%, *p* = 0.002; TT vs. CC: I^2^ = 70.3%, *p* = 0.009; TC vs. CC: I^2^ = 84.0%, *p* = 0.000; TT + CT vs. CC: I^2^ = 85.8%, *p* = 0.000, respectively), the result showed no significant association between *IGF-1* rs35767 and T2DM risk with random effects model (T vs. C: OR = 1.065, 95% CI: 0.983–1.153, *p* = 0.126; TT vs. CC: OR = 1.603, 95% CI: 0.996–2.578, *p* = 0.052; TC vs. CC: OR = 1.407, 95% CI: 0.937–2.112, *p* = 0.099; TT + CT vs. CC: OR = 1.469, 95% CI: 0.978–2.207, *p* = 0.064, respectively) (Figure 4). Since there are not sufficient evidence to draw the conclusion that the rs35767 SNP is associated with T2DM.

### 3.4 Subgroup-Analyses

**FIGURE 5:**
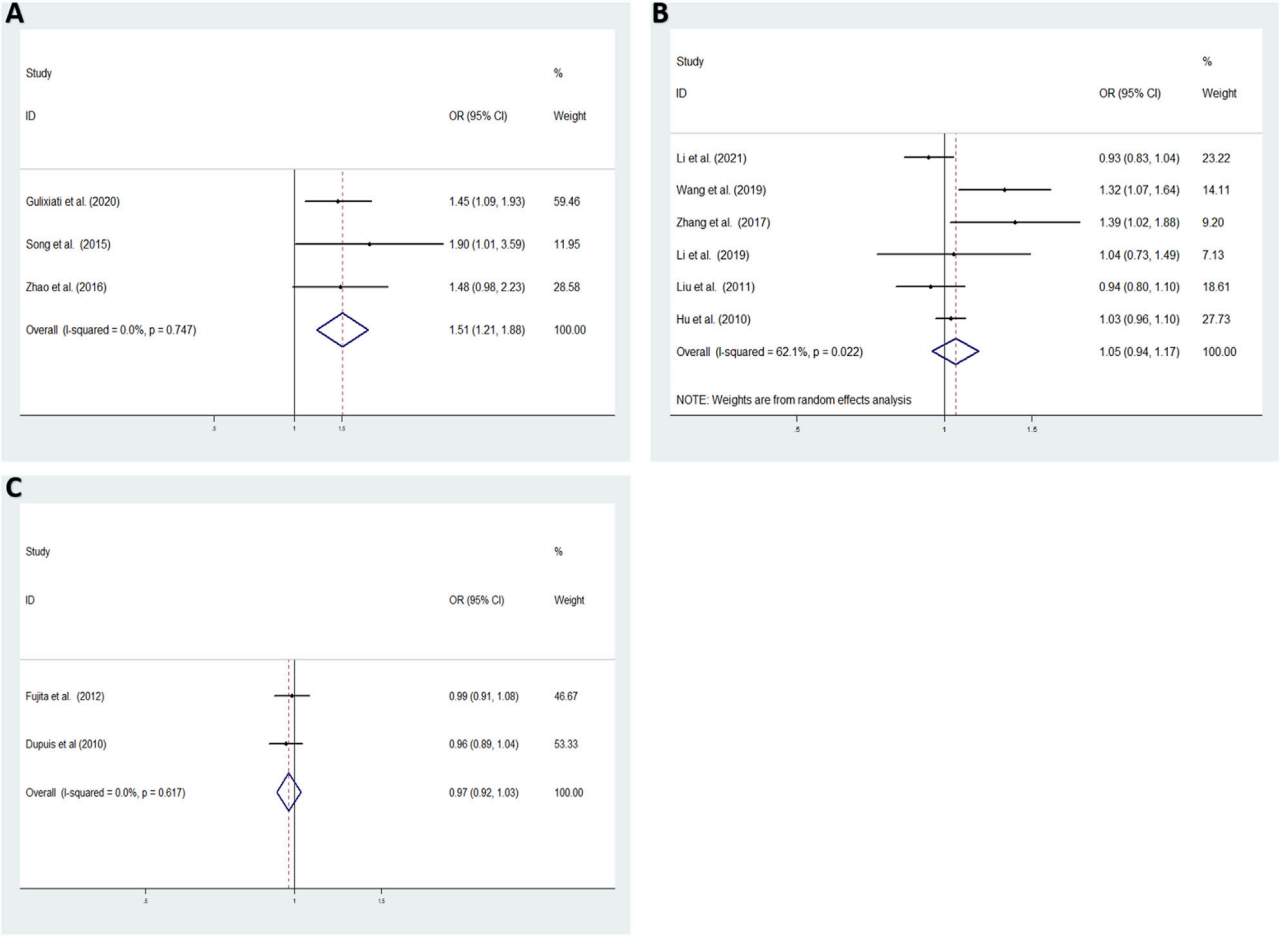
The association between the *IGF-1* rs35767 and T2DM susceptibility in the subgroup for the allele model (T vs. C) **(A)** *XinJiang*, China (fixed effects model) **(B)** *Other provinces*, China (random effects model) **(C)** Other countries (fixed effects model). OR: odds ratio, CI: confidence interval, I-squared: measure to quantify the degree of heterogeneity in meta-analyses.

As high heterogeneity was observed, we performed subgroup-analysis according to origin to evaluate the association between rs35767 and T2DM susceptibility in the allele model. The results suggested that rs35767 was significantly related to the risk of T2DM in *XinJiang*, China subgroup (T vs. C: OR = 1.508, 95% CI: 1.210–1.878, *p* = 0.000) with fixed effects model; a random effects model were used to analyze the other provinces, China, rs35767 was shown no significant association with T2DM risk (T vs. C: OR = 1.051, 95% CI: 0.943–1.173, *p* = 0.369); and not a significantly associatied in the other countries subgroup (excluding Gouda et al. Literature) (T vs. C: OR = 0.975, 95% CI: 0.922–1.031, *p* = 0.376) with fixed effects model (Figure 5).

### 3.5 Publication Bias

The funnel plot was showed to be visually symmetrical (Supplementary Figure S1, Supplementary Figure S2, Supplementary Figure S3). Begg’s and Egger’s tests were performed to detect publication bias. There was no significant publication bias appeared in all genetic models in overall analysis *via* Begg’s test (all *p* > 0.05, Supplementary Table S2), but for Egger’s test, there was publication bias in the additive model (heterozygote comparisons) (Egger’s test, *p* = 0.006, Supplementary Table S2). We did not determine publication bias for Begg’s test after omitting Gouda’s study and subgroup analysis in all genetic models (all *p* > 0.05, Supplementary Table S3, Supplementary Table S4). However, for Egger’s test, there were publication bias in the allele model (Egger’s test, *p* = 0.039, Supplementary Table S3) and additive model (heterozygote comparisons) (Egger’s test, *p* = 0.031, Supplementary Table S4).

## 4 Discussion

Previous studies have showed the inconsistent findings of association between *IGF-1* rs35767 and the risk of T2DM. Gouda et al. revealed that the TT, TT + CT genotypes of rs35767 were associated with an increased risk of T2DM in pregnant Egyptian women respectively (Gouda et al., 2019). This results were successfully replicated in a Chinese Han population (Wang. 2019). Zhang et al. found that the A allele of rs35767 contributed to the risk of developing T2DM in a Chinese Han population (Zhang.et al., 2017). More recently, two studies documented that the association of the rs35767 in *IGF-1* was associated with T2DM in a Uyghur population in China (GulixiatiMaimaitituersun. 2020; Song et al., 2015). However, some studies did not find evidence of an association between rs35767 and T2DM (Dupuis et al., 2010; Hu et al., 2010; Fujita et al., 2012; Liu et al., 2012; Zhao et al., 2016; Li et al., 2019; Li et al., 2021). It is worth noting that the four largest studies with the most statistical power (Dupuis et al., Hu et al., Fujita et al., and Li et al., 2021) did not report statistically significant associations between the rs35767 SNP and T2DM, whereas five small studies (Song et al., Zhang et al., Wang et al., Gouda et al., and Gulixiati et al.) all reported statistically significant associations. Compared the subjects selected for the studies between the larger and smaller studies, we found that in the Gouda et al. study, the mean body mass index (BMI) of subjects with T2DM was 34.26 ± 5.7, which is very different from a mean BMI of 26.96 ± 4.57 for control subjects without T2DM. There were a significant difference concerning BMI between T2DM and control groups. This study included only pregnant women, was observed to be very influential on the initial analyses.

Previous studies have been reported that people with a low *IGF-1* level are prone to have diabetes mellitus (Colao et al., 2013; Shankar and Li, 2013). The functional SNP rs35767 (T > C) in *IGF-1* promoter, with C allele showed a higher transcriptional activity than promoter with T allele (Telgmann et al., 2009). In terms of mechanism, the higher transcriptional activity of the C allele *IGF-1* promoter was contributed by the C/EBPD transcription activator, which bound exclusively to the C allele, but not to the T allele (Chen et al., 2013; Telgmann et al., 2009). Therefore, there may have low *IGF-1* expression level when promoter with rs35767 T allele, which contribute to the development of T2DM. In our meta-analysis, we found that T allele, TT genotype, TT + CT genotype of rs35767 increased T2DM risk in overall analysis, as well as increasing the risk of T2DM in Uyghur population. After omitting Gouda’s study, the result showed TT genotype were of borderline statistical significance.

In overall analysis, high heterogeneity among studies were detected in four genetic models, which might be a result of the difference in ethnicity, country, genetic background and environmental factors (e.g., dietary, life style, climates) (Qin et al., 2010). Then omittied Gouda’s study, the heterogeneity was reduced. We found that the subjects of Gouda’s study were pregnant Egyptian women, but participants of other studies were both male and female. Thus gender ratio may also had a certain impact on heterogeneity. The subgroup-analyses were detected by origin in allele model, the subgroup of Uyghur in Xinjiang, China have no heterogeneity, but other subgroups still had high heterogeneity. It is noteworthy that a previous study in Xinjiang found that 19.6% of Uyghur had diabetes, exceptionally higher than that in Kazakh (7.3%) and Han Chinese (9.1%) (Li et al., 2012; Song et al., 2015). The marriage pattern and unique life style might be responsible for the observation. One hand, the practice of endogamy in Uyghur population might also be a reason (Wang et al., 2003; Mamet et al., 2005). On the other hand, The most Uyghurs have different dietary habits from Han Chinese. They have more meat, high carbohydrate diets with a higher salt (more than 20 g per day) and less unsaturated fatty acids compared with Han Chinese (Zhai et al., 2007).

There still have several limitations in our meta-analysis. Firstly, there were limited studies which estimated *IGF-1* rs35767 and T2DM risk, only six articles had four gene models data, and the other six articles had only one allele model data. Secondly, the results did not adjustment the potential risk factors, including gender, body mass index, age, drinking and smoking status, and environmental factors. Thirdly, some results showed relatively obvious heterogeneity, but research the source of heterogeneity needs to more larger sample. Finally, some groups results existed potential publication bias in Egger’s test. Therefore, the results of the article should be interpreted carefully.

## 5 Conclusion

This is the first time to perform a meta-analysis to systematically summarize the association between the *IGF-1* rs35767 and T2DM susceptibility. Overall, there is not enough evidence from the results of the meta-analysis to indicate that the rs35767 SNP has a statistically significant association with T2DM. Further more studies are necessary to verify the results.

## Data Availability

The original contributions presented in the study are included in the article/Supplementary Material further inquiries can be directed to the corresponding authors.
