# Supplementary figures and images for: Association Between Insulin-like Growth Factor-1 rs35767 Polymorphism and Type 2 Diabetes Mellitus Susceptibility: A Meta-Analysis
Source: Front Genet. 2021 Nov 22;12:774489. doi: 10.3389/fgene.2021.774489 (PMC8646032; doi:10.3389/fgene.2021.774489)

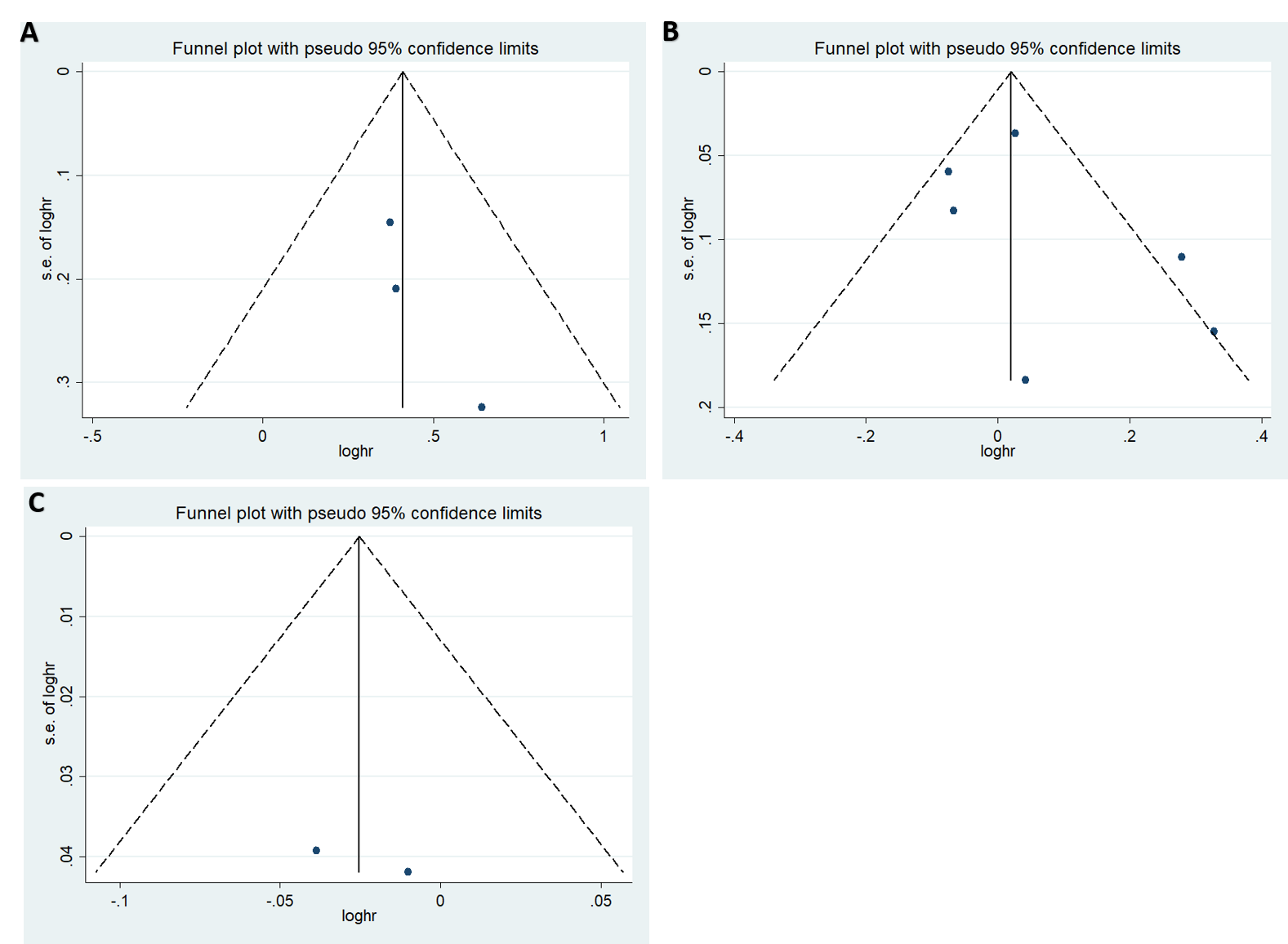

Supplement: Supplementary file 2 [file Image3.TIF]

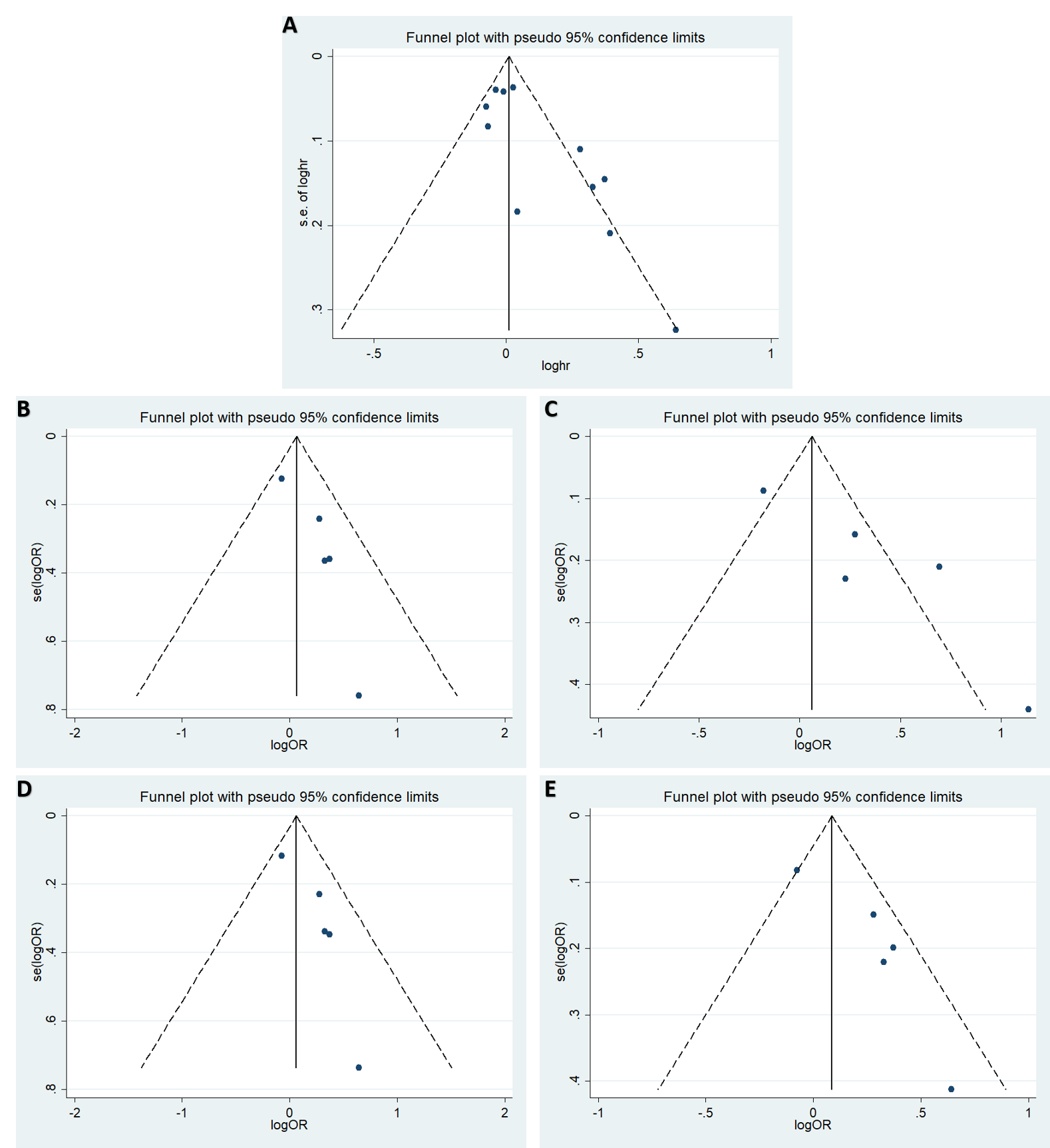

Supplement: Supplementary file 3 [file Image2.TIF]

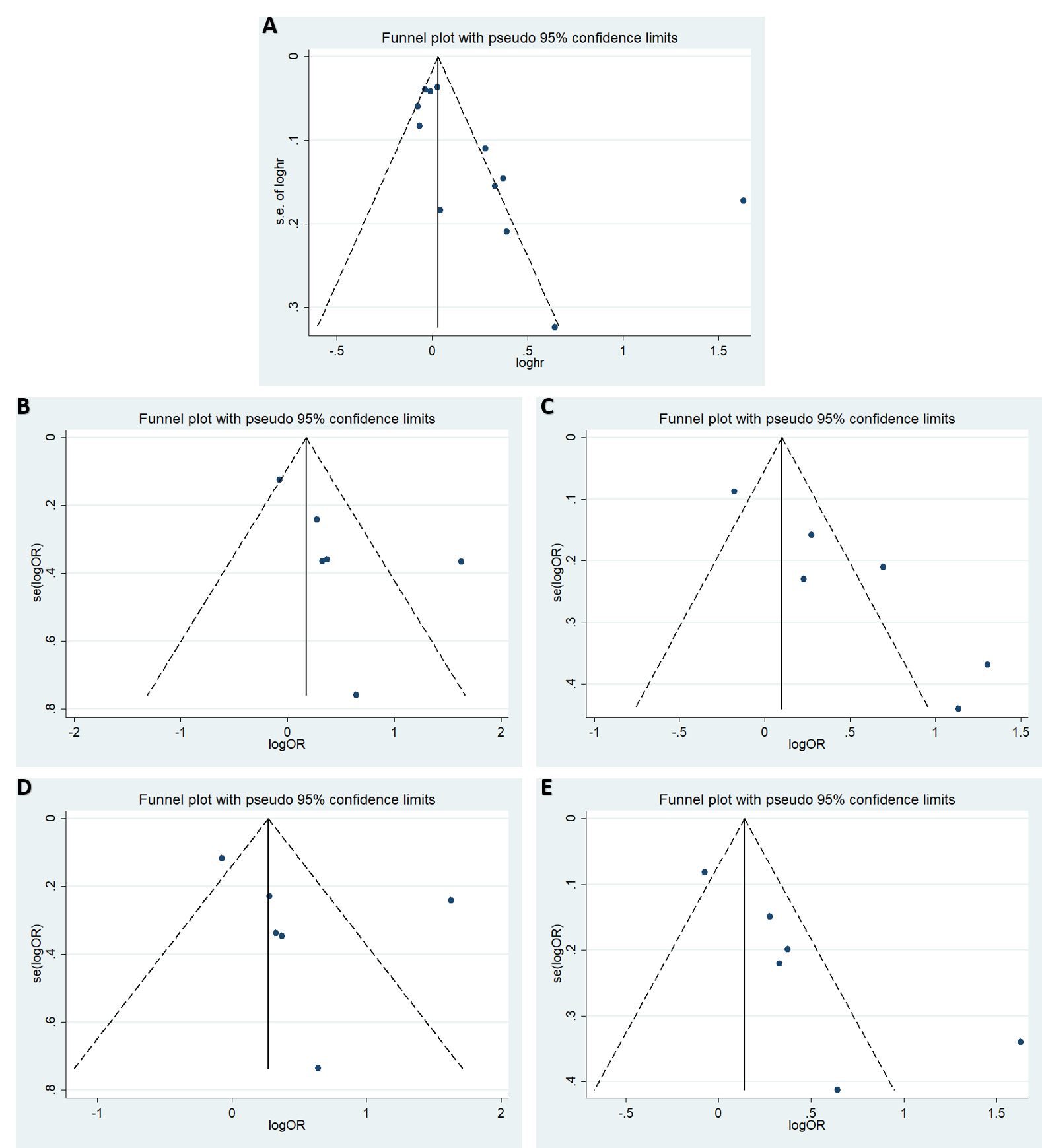

Supplement: Supplementary file 4 [file Image1.TIF]
